# Subchronic Pulmonary Pathology, Iron Overload, and Transcriptional Activity after Libby Amphibole Exposure in Rat Models of Cardiovascular Disease

**DOI:** 10.1289/ehp.1103990

**Published:** 2011-10-06

**Authors:** Jonathan H. Shannahan, Abraham Nyska, Mark Cesta, Mette C.J. Schladweiler, Beena D. Vallant, William O. Ward, Andrew J. Ghio, Stephen H. Gavett, Urmila P. Kodavanti

**Affiliations:** 1 Curriculum in Toxicology, University of North Carolina School of Medicine, Chapel Hill, North Carolina, USA; 2 Sackler School of Medicine, Tel Aviv University, Timrat, Israel; 3 Cellular and Molecular Pathology Branch, National Institute of Environmental Health Sciences, National Institute of Health, Research Triangle Park, North Carolina, USA; 4 Cardiopulmonary and Immunotoxicology Branch, Environmental Public Health Division; 5 Genomics Core, and; 6 Biostatistics Core, Research Core Units, National Health and Environmental Effects Research Laboratory, Office of Research and Development, U.S. Environmental Protection Agency, Research Triangle Park, North Carolina, USA; 7 Clinical Research Branch, Environmental Public Health Division, National Health and Environmental Effects Research Laboratory, Office of Research and Development, U.S. Environmental Protection Agency, Chapel Hill, North Carolina, USA

**Keywords:** cardiovascular disease, iron overload, Lilly amphibole, pulmonary pathology

## Abstract

Background: Surface-available iron (Fe) is proposed to contribute to asbestos-induced toxicity through the production of reactive oxygen species.

Objective: Our goal was to evaluate the hypothesis that rat models of cardiovascular disease with coexistent Fe overload would be increasingly sensitive to Libby amphibole (LA)-induced subchronic lung injury.

Methods: Male healthy Wistar Kyoto (WKY), spontaneously hypertensive (SH), and SH heart failure (SHHF) rats were intratracheally instilled with 0.0, 0.25, or 1.0 mg LA (with saline as the vehicle). We examined bronchoalveolar lavage fluid (BALF) and histological lung sections after 1 week, 1 month, or 3 months for pulmonary biomarkers and pathology. SHHF rats were also assessed at 6 months for pathological changes.

Results: All animals developed concentration- and time-dependent interstitial fibrosis. Time-dependent Fe accumulation occurred in LA-laden macrophages in all strains but was exacerbated in SHHF rats. LA-exposed SHHF rats developed atypical hyperplastic lesions of bronchiolar epithelial cell origin at 3 and 6 months. Strain-related baseline differences existed in gene expression at 3 months, with persistent LA effects in WKY but not SH or SHHF rats. LA exposure altered genes for a number of pathways, including inflammation, immune regulation, and cell-cycle control. Cell-cycle control genes were inhibited after LA exposure in SH and SHHF but not WKY rats, whereas tumor suppressor genes were induced only in WKY rats. The inflammatory gene expression also was apparent only in WKY rats.

Conclusion: These data show that in Fe-overload conditions, progressive Fe accumulation occurs in fiber-laden macrophages within LA-induced lesions. Fe overload does not appear to contribute to chronic inflammation, and its role in hyperplastic lesion development requires further examination.

During vermiculite mining, residents and miners in Libby, Montana (USA), were exposed to a mixture of amphibole-type asbestos fibers containing winchite, richerite, tremolite, and other minerals (Moolgavkar et al. 2010). Epidemiological studies have shown a dose-dependent correlation between Libby amphibole (LA) exposure and the incidences of asbestos-induced diseases, including asbestosis, lung cancer, and mesothelioma (Moolgavkar et al. 2010). Animal studies have shown that the toxicity of amphibole fibers is possibly due to their biopersistence within the lung, causing prolonged inflammation and oxidative stress (Bernstein et al. 2003).

Asbestos produces reactive oxygen species (ROS) through Fenton reactions likely facilitated by surface-available metals such as iron (Fe). Asbestos and silica can associate with endogenous Fe after inhalation, forming Fe-rich asbestos bodies on fiber surfaces (Ghio et al. 2004). After binding, this Fe is postulated to enhance the ability of asbestos to produce ROS, thereby exacerbating its toxicity (Hardy and Aust 1995). We have shown that LA can bind Fe and that this Fe is redox active in an acellular environment (Shannahan et al. 2011a). However, Fe loading of LA or cells inhibits the acute pro-inflammatory response *in vitro* in cells and *in vivo* in rats (Shannahan et al. 2011a). The role of Fe in the progression of the diseases induced by LA and other asbestos materials is unknown.

Increased tissue Fe overload may increase bioactive and protein-bound Fe levels and affect the inflammatory response after an environmental insult. Individuals with chronic diseases such as thalassemia, diabetes, arthritis, cancer, and cardiovascular disease (CVD) have been shown to have systemic Fe overload (Abdalla et al. 2011; Sen et al. 2011; Toyokuni 2011). The systemic Fe overload, associated inflammation, and oxidative stress in CVD (Kruszewski 2004) may influence host susceptibility to asbestos-induced diseases. We have shown that the spontaneously hypertensive (SH) and SH heart failure (SHHF) rats exist in a state of pulmonary Fe overload (as demonstrated by increased pulmonary ferritin and transferrin), inflammation, and oxidative stress compared with control Wistar Kyoto (WKY) rats (Shannahan et al. 2010). The hypertensive state of both the SH and SHHF rats has been linked to genetic anomalies in the rennin angiotensin system (Leckie 2001) and the β-adrenergic system (Remmers et al. 1993); however, the role of other genetic factors remains unknown. Previous work has shown that an acute exposure to LA in these rat models does not exacerbate inflammation despite increases in Fe-binding proteins and gene expression (Shannahan et al. 2011b). High baseline inflammation together with dysregulated Fe homeostasis might diminish the acute inflammogenic response to toxicants. In the present study, we hypothesized that the subchronic presence of LA in the lungs of SH and SHHF rats will cause further accumulation of fiber-associated Fe and inhibit inflammation. Furthermore, we presumed that examining the transcriptional activity of the whole lung genome might provide additional insights into the role of Fe in the progression of asbestos-induced lung disease.

## Materials and Methods

*Animals.* Male, 11- to 12-week-old, healthy WKY, SH, and obese SHHF rats were purchased from Charles River Laboratories (Raleigh, NC, USA). Rats were maintained in an Association for Assessment and Accreditation of Laboratory Animal Care–approved animal facility at 21 ± 1°C, 50 ± 5% relative humidity, and 12/12-hr light/dark cycle. Animals received standard rat chow (Rat Chow 5001; Purina Mills, Brentwood, MO, USA) and water *ad libitum*. The Institutional Animal Care and Use Committee of the U.S. Environmental Protection Agency’s National Health Environmental Effects Research Laboratory approved the protocol. The animals were treated humanely and with regard for alleviation of suffering.

*Intratracheal instillation of Libby amphibole.* The Libby amphibole (LA) sample collected from the Rainy Creek complex near Libby, Montana, in 2007 was size fractionated to isolate a rat respirable fraction (particulate matter ≤ 2.5 μm in aerodynamic diameter (i.e., PM_2.5_). Rats (WKY, *n* = 12/time point; SH, *n* = 6/time point; SHHF, *n* = 6/time point) were intratracheally instilled with saline or 0.25 or 1.0 mg of LA (with saline as the vehicle) as described previously (Shannahan et al. 2011a) [also see “Methods” in Supplemental Material (http://dx.doi.org/10.1289/ehp.1103990)]. Doses were chosen to assure a response in the lung, allowing for a comparative analysis between strains. Theoretically, a rat will deposit 0.07 mg fibers during 6 hr of inhalation at 10 mg/m^3^, assuming that minute volume is 200 mL and the deposition fraction to pulmonary region is 0.10. The WKY sample size was increased to twice that of SH and SHHF rats so that data from WKY rats with nonpathological cardiac hypertrophy could be excluded for comparisons (Shannahan et al. 2010). An additional experiment was also performed in which 36 SHHF rats were exposed to saline or 0.25 or 1.0 mg LA in saline and examined at 3 or 6 months for bronchoalveolar lavage fluid (BALF) and lung pathology.

*Necropsy, sample collection, and analysis.* Rats were anesthetized with sodium pentobarbital (Virbac AH, Fort Worth, TX, USA; 50–100 mg/kg, intraperitoneal) 1 week, 1 month, or 3 months after instillation of LA. Blood was removed from the vasculature of the lung by perfusion via the pulmonary artery to avoid interference in data analysis involving Fe-binding proteins. The right lung lobes were lavaged with Ca^2+^/Mg^2+^-free phosphate-buffered saline and stored at –80°C for later analysis. The left lung was fixed by instillation of 10% neutral buffered formalin through the trachea.

*Cell differential and analysis.* Aliquots of BALF were taken for total cell counts (Coulter, Miami, FL, USA). Differential cell determinations of cytospin preparations (Shandon, Pittsburgh, PA, USA) were made using LeukoStat staining (Fisher Scientific, Pittsburgh, PA, USA). Macrophages and neutrophils were counted under light microscopy. Cell-free BALF was analyzed for total protein, albumin, ferritin, and transferrin (Shannahan et al. 2010) [see Supplemental Material, “Methods” (http://dx.doi.org/10.1289/ehp.1103990)].

*Lung histopathology.* The left lung was embedded in paraffin, sectioned to a thickness of 3 μm (transverse), and stained with hematoxylin and eosin (H&E), Perls Prussian blue, or Masson’s trichrome (Experimental Pathology Labs, Durham, NC, USA). Histopathological changes in the tissues were scored using semiquantitative grading at five levels (0, normal; 1, minimal; 2, mild; 3, moderate; 4, severe) taking into consideration the degree of severity and the type of lesion [see Supplemental Material, “Methods” (http://dx.doi.org/10.1289/ehp.1103990)]. In an additional experiment, lung tissues from SHHF rats were stained with H&E and examined for lesions at 3- and 6-month time points.

*Immunohistochemistry.* The cellular origin of hyperplasic changes in SHHF rats at 3 months was examined immunohistochemically. Five-micrometer-thick lung sections from rats exposed to 0.0, 0.25, or 1.0 mg LA for 3 months were immunohistochemically stained for several epithelial cell markers [see Supplemental Material, “Methods” (http://dx.doi.org/10.1289/ehp.1103990)]. Sections were assessed for surfactant protein A (sc-7699), Clara cell protein 10 (sc-9772), cytokeratin 10 (sc-58720), and cytokeratin 14 (sc-53253) using antibodies from Santa Cruz Biotechnology (Santa Cruz, CA, USA) and cytokeratin 19 (CRC946) from Cell Marque (Rocklin, CA, USA).

*Gene array.* Total RNA was isolated from caudal lung lobes using RNeasy Mini Kits (Qiagen, Valencia, CA, USA). RNA integrity was assessed by the RNA 6000 LabChip® (Caliper Life Sciences, Hopkinton, MA, USA) kit using an Agilent 2100 bioanalyzer (Agilent Technologies, Palo Alto, CA, USA). Changes in global gene expression were examined at the 3-month time point in each strain for saline control and LA (1.0 mg/rat) groups (*n* = 6) using the Affymetrix platform (Rat Genome 230 2.0 Array; Affymetrix, Santa Clara, CA, USA) [see Supplemental Material, “Methods” (http://dx.doi.org/10.1289/ehp.1103990)].

*Statistical analysis of BALF and pathology data.* Data are expressed as mean ± SE (WKY, *n* = 8–12/group; SH and SHHF, *n* = 6/group). SigmaStat (version 3.5; Systat Software, Point Richmond, CA, USA) was used to determine statistical comparisons via a two-way analysis of variance (ANOVA) followed by a post hoc comparison using the Holm-Sidak method (*p* ≤ 0.05). Subjective numerical scoring of Perls blue and trichrome staining assigned to each animal were added together for each group and statistically analyzed using Fisher’s exact test. Statistical significance was determined when *p* ≤ 0.05.

*Gene array data analysis.* The CEL files were loaded into Rosetta Resolver (version 7.2; Rosetta Inpharmatics, Seattle, WA, USA) and normalized with the Rosetta error model. Differentially expressed probe sets were determined using a one-way ANOVA with a Benjamini-Hochberg multiple-test correction (false discovery rate set at 0.05). Each of the five treatments (including SH saline and SHHF saline) was contrasted with WKY saline. The resulting five lists of differentially expressed genes (DEGs) were combined to generate a master list. A Venn diagram was generated from the contrast of LA-induced changes relative to control for each strain using Rosetta Resolver.

*Functional analysis of gene array data set.* For a global assessment of affected Kyoto Encyclopedia of Genes and Genomes (KEGG) pathways (Bioinformatics Center, Institute of Clincial Research, Kyoto University, Kyoto, Japan; http://www.genome.jp/kegg/), the master list of DEGs relative to WKY saline was submitted to DAVID (Database for Annotation, Visualization and Integrated Discovery; http://david.abcc.ncifcrf.gov/) Huang et al. 2009a, 2009b) for analysis. *p*-Values using a Fisher exact test indicate overrepresentation of DEGs in a pathway and were used to generate a heat map of KEGG pathways. Functional lists of genes were constructed from KEGG pathway genes for inflammation and cell-cycle control [see Supplemental Material, “Methods” (http://dx.doi.org/10.1289/ehp.1103990)]. Each functional list is depicted in a heat map, where each gene was median centered considering individual animals in all six groups and clustered based on commonality of directional changes. Eisen’s cluster analysis was used for clustering genes (Eisen et al. 1998) and the results were viewed using Treeview [Cluster version 3.0; Michiel de Hoon (mdehoon@ims.utokyo.ac.jp); University of Tokyo, Human Genome Center, Tokyo, Japan].

## Results

**Figure 1:**
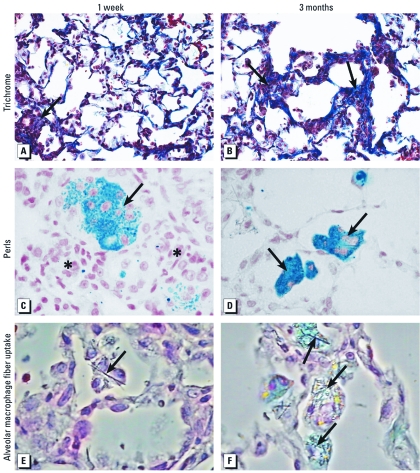
Time-related collagen deposition, ferric Fe accumulation, and alveolar macrophage fiber uptake in the lung after a single intratracheal instillation of LA in SHHF rats: photomicrographs of lung tissue sections 1 week (left column) and 3 months (right column) after exposure to 1.0 mg LA. (*A*,*B*) Stained for collagen using Masson’s trichrome. Arrows indicate blue staining in fibrotic lesions. Magnification, 20×. (*C,D*) Stained for ferric Fe (arrows) using Perls blue. The asterisks indicate unstained fibrotic areas. Magnification, 60×. (*E*,*F*) Perls blue–stained sections of lung using phase-contrast microscopy to focus on alveolar macrophage uptake of LA fibers. Arrows indicate fiber locations. Magnification, 40×.

*Lung injury markers, histopathological evaluation, and ferric Fe staining.* We noted baseline strain-related differences in BALF markers of inflammation and lung injury; however, no LA-inducible changes were apparent at 3 months after exposure [see Supplemental Material, Figure 1 (http://dx.doi.org/10.1289/ehp.1103990)]. BALF changes and lung histological analysis for the 1-day, 1-week, and 1-month time points have been reported recently (Shannahan et al. 2011b). In the present study, we examined pathological lung lesions from 3-month H&E slides and also performed trichrome staining for collagen and Perls blue staining for ferric Fe at 1 week, 1 month, and 3 months in order to understand the time course of the development of fibrosis and accumulation of ferric Fe. All strains demonstrated interstitial fibrosis after exposure to LA (Table 1). More intense fibrotic foci were apparent in terminal bronchiolar areas where fibers likely accumulate (Figure 1A,B). No strain-related differences could be ascertained from analyzing the trichrome staining. BALF and histological lung sections from control WKY and SH rats did not stain for ferric Fe, whereas a few alveolar macrophages stained positive in control SHHF rats (Table 1). No discernible changes in Perls blue staining were apparent in LA-exposed WKY rats at any time point. At 1 month and 3 months, only a few macrophages stained positive for ferric Fe in SH rats exposed to 1 mg LA. The SHHF rats had markedly greater numbers of macrophages that stained positive for Fe compared with WKY and SH rats (SHHF > SH > WKY; Table 1). These Fe-positive macrophages were observed around the lesions of interstitial fibrosis (Figure 1C,D). Alveolar macrophages appeared to internalize LA by 1 week after exposure, and fibers remained visible in macrophages at 3 months after expsoure (Figure 1E,F).

**Table 1:** Histological evaluation of relative staining intensities for ferric Fe and collagen after exposure to LA in WKY, SH, and SHHF rats: average score and number of animals affected by exposure.

Average score (no. of animals affected by exposure)						
Time point	LA concentration (mg/rat)	Animals/group	Perls blue staining for ferric Fe	Trichrome staining for collagen
One week								
WKY		0.0		5		0.0		0.0
		0.25		6		0.0		1.0 (6)*
		1.0		6		0.3 (2)		1.0 (6)*
SH		0.0		6		0.0		0.2 (1)
		0.25		6		0.0		1.0 (6)*
		1.0		6		0.3 (2)		1.0 (6)*
SHHF		0.0		6		0.0		0.0
		0.25		6		0.8 (4)*^#^		1.0 (6)*
		1.0		6		0.8 (4)*		1.2 (6)*
One month								
WKY		0.0		5		0.0		0.0
		0.25		5		0.0		1.0 (5)*
		1.0		5		0.0		0.8 (4)*
SH		0.0		6		0.0		0.0
		0.25		6		0.0		0.8 (5)*
		1.0		5		1.0 (4)*^#^		1.0 (6)*
SHHF		0.0		6		0.3 (2)		0.0
		0.25		6		1.5 (6)*^#^		1.0 (6)*
		1.0		6		1.7 (6)*^#^		1.2 (6)*
Three months								
WKY		0.0		5		0.0		0.0
		0.25		6		0.0		1.0 (6)*
		1.0		5		0.4 (2)		1.7 (5)*
SH		0.0		6		0.0		0.0
		0.25		6		0.5 (2)		0.8 (5)*
		1.0		6		0.8 (3)*		1.5 (6)*
SHHF		0.0		6		0.3 (2)		0.0
		0.25		5		0.6 (2)		0.8 (5)*
		1.0		6		1.7 (6)*^#^		2.0 (6)*
WKY, SH, and SHHF rats exposed to saline (control) or LA (0.25 or 1.0 mg/rat) were examined for pulmonary collagen deposition and ferric Fe accumulation using Masson’s trichrome and Perls blue staining, respectively. Values represent average severity score, from 0 to 4 in increasing severity. *p < 0.05, Fisher’s exact test, within strain compared with saline control. ^#^*p* < 0.05, strain difference compared with WKY at the same exposure concentration.								

**Figure 2:**
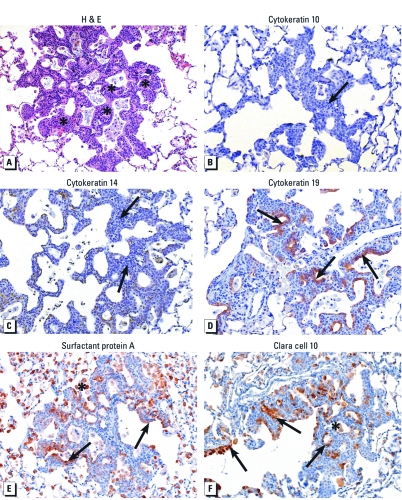
Immunohistochemical characterization of atypical hyperplastic lesions in SHHF rats at 3 months after a single instillation of 1.0 mg LA. (*A*) H&E-stained lung section. Asterisks indicate the locations of hyperplastic lesions. (*B*, *C*) Lung sections immunohistochemically stained for the squamous cell markers cytokeratin 10 (*B*) and cytokeratin 14 (*C*). The hyperplastic changes (arrows) did not stain for cytokeratins 10 and 14. (*D*) Lung sections stained for the bronchiolar cell marker cytokeratin 19 (brown staining shown by arrows). (*E*, *F*) Lung sections stained for surfactant protein A (*E*), a marker of alveolar type II and Clara cells, and the Clara cell marker Clara cell 10 (*F*). Arrows indicate locations of epithelial cells; asterisks indicate areas of staining in macrophages. Negative control antibodies did not stain positive for any marker. Magnification, 20×.

One important observation made in 3-month H&E-stained slides of LA-exposed SHHF rats was the presence of focal atypical hyperplastic lesions (Figure 2A). These lesions appeared to be concentration dependent [saline, 0/6 lesion incidence (no. lesions/no. rats from which lung sections were stained with H&E); 0.25 mg LA, 2/6 lesion incidence; 1 mg LA, 4/6 lesion incidence]. The lesions were located at or near terminal bronchioles and were characterized by stacked, multiple layers of epithelial cells lining alveolar ducts or alveolar walls. To determine the further progression of hyperplastic changes over time, additional SHHF rats instilled at the same dose levels were examined 3 and 6 months after LA exposure. This experiment verified the presence of atypical hyperplastic lesions in SHHF rats at 3 and 6 months after LA exposure; however, the lesions did not progress in severity from 3 to 6 months (data not shown).

*Immunohistochemical evaluation.* In order to determine the cellular origin of these hyperplastic lesions and the possible presence of squamation, the tissue sections were immunohistochemically evaluated using several cytokeratin and epithelial cell markers in SHHF rats. These lesions were negative for the squamous epithelial markers cytokeratin 10 and 14, as indicated by a lack of brown staining (Figure 2B,C). However, lesions stained positive (brown stained cells) for *a*) the bronchiolar epithelial cell marker cytokeratin 19 (Figure 2D), *b*) the Clara cell and alveolar type II cell marker surfactant protein A (Figure 2E), and *c*) the Clara cell–specific marker Clara cell 10 (Figure 2F). These results are consistent with a bronchiolar epithelial cell origin of the hyperplastic lesions.

*Gene expression analysis.* We compared the gene expression profiles of controls with those of rats treated with 1.0 mg LA in each strain to determine which genes and pathways differed at baseline and remained altered at 3 months. Baseline strain differences primarily involved genes related to immune regulation, inflammation, and metabolic disease condition. It was apparent from the Venn diagram [see Supplemental Material, Figure 2 (http://dx.doi.org/10.1289/ehp.1103990)] that nearly 2,000 genes had changed in the WKY and SH rats at 3 months after LA exposure, whereas minimal changes (180 genes) occurred in the SHHF rats. Only 15 genes were modified in common among all three strains at 3 months after LA exposure. The SH rats demonstrated the highest total number of genes changed after LA exposure (SH > WKY > SHHF), with more genes having down-regulated compared with the WKY and SHHF rats.

**Figure 3:**
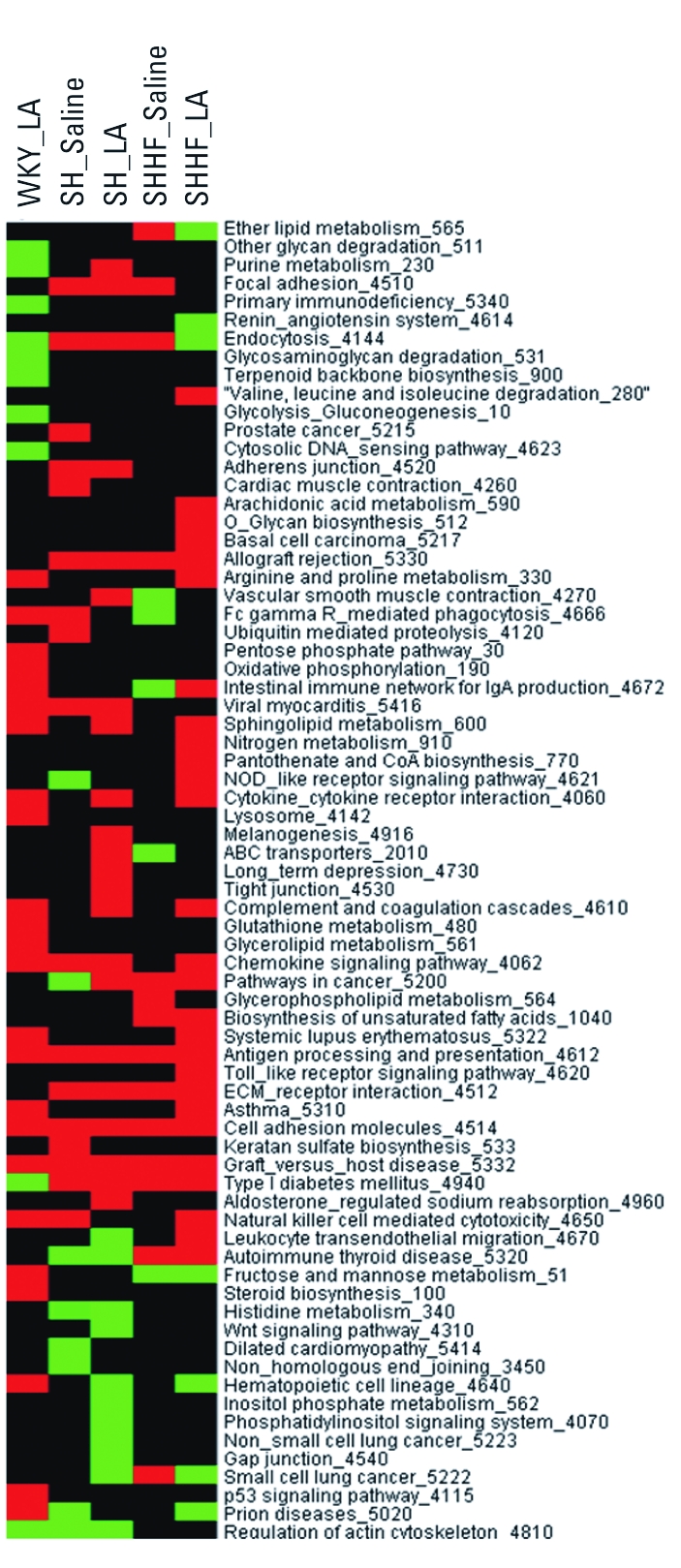
Heat map of KEGG pathways showing significant difference in LA-exposed WKY, SH, and SHHF rats and control SH and SHHF rats compared with control WKY rats. Black indicates that no genes in the pathway have altered transcription compared with control (WKY saline). Green indicates that the pathway has too few genes with altered transcription compared with control (WKY saline) to be significant by the Fisher exact test. Red indicates that the pathway contains a sufficient number of transcriptionally altered genes to be significant (*p* ≤ 0.05) by the Fisher exact test.

In order to understand the relative strain differences and LA effects in parallel, we compared the mean expression values of WKY saline control rats with those of WKY LA, SH saline, SH LA, SHHF saline, and SHHF LA rats and determined relative fold differences. Although we found marked intensity differences after LA exposure, most of the baseline gene changes seen in SH and SHHF rats compared with WKY rats were in the same direction (up or down) as those seen in WKY rats. The pathways significantly altered because of baseline strain differences or LA exposure, based on the list of DEGs for each contrast, are depicted in the heat map shown in Figure 3.

Several KEGG pathways, including those involving lipid and steroid metabolism, glycolysis, inflammation, leukocyte migration, cell-cycle control, oxidative phosphorylation, and diabetes, were found altered at baseline and at 3 months after exposure to LA (Figure 3). The glutathione metabolism pathway was significantly changed only in WKY rats exposed to LA. Similarly, only WKY rats exposed to LA demonstrated an effect on pathways related to oxidative phosphorylation and tumor suppression (p53) signaling. SH rats exposed to LA demonstrated an effect on pathways assigned to cancer, whereas SHHF rats had this pathway affected significantly at baseline as well as after LA exposure (Figure 3). SHHF rats exposed to LA also had other pathways related to cell proliferation induced, including basal cell carcinoma.

**Figure 4:**
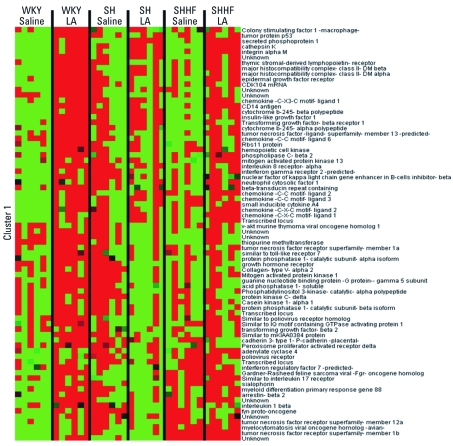
Hierarchical clustering of inflammation genes comparing WKY, SH, and SHHF rats at 3 months after saline or 1.0 mg LA exposure. DEGs related to inflammation were assembled from KEGG pathways 4060, 4062, 4070, 4310, 4510, 4514, 4520, 4620, 4621, and 4670. Clusters 1 and 2 with genes up-regulated in WKY rats exposed to LA were separated [cluster 2 heat map is shown in the Supplemental Materials, Figure 3 (http://dx.doi.org/10.1289/ehp.1103990)]. Red indicates genes that have high expression values, green indicates genes that have low expression values across all groups, and black indicates median expression.

Hierarchical clustering within selected KEGG pathways was performed to understand the strain-related directional changes in gene expression belonging to specific processes. As indicated in Figure 4, many genes related to inflammation were induced at baseline in SH rats and to some degree in SHHF rats compared with WKY controls. The expression levels of these same genes were increased more readily after LA exposure in WKY rats, and to a lesser extent in SHHF rats, but had a tendency to be inhibited in SH rats. Notable examples included genes for nuclear factor κB (*NF*κ*B*), tumor necrosis factor, and a variety of interleukins. Genes expressed at high levels in WKY rats at baseline compared with baseline in SH and SHHF rats are shown in cluster 2 [see Supplemental Material, Figure 3 (http://dx.doi.org/10.1289/ehp.1103990)]. These genes remained unchanged in WKY rats but tended to be inhibited from their already low baseline in SH and SHHF rats after LA exposure. These genes included those for chemokines such as *CXCL-14* [chemokine (C-X-C motif) ligand 14] and CCL-5 [chemokine (C-C motif) ligand 5], suggesting that some genes that regulate inflammatory response to LA tend to be inhibited in SH and SHHF rats but not in WKY rats.

**Figure 5:**
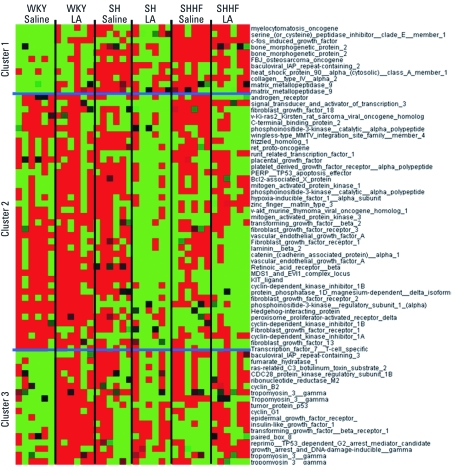
Clustering of cell-cycle control–specific pulmonary genes comparing WKY, SH, and SHHF rats 3 months after exposure of 1.0 mg LA. DEGs related to cell-cycle control were assembled from KEGG pathways 4115 and 5200. All genes were hierarchically clustered, median centered, and viewed using Treeview as indicated in Figure 4.

Strain differences at baseline and after LA exposure were noted in the genes involved in cell-cycle control (Figure 5). Genes expressed at high levels in the lungs of control SH and SHHF rats, including the gene for matrix metalloproteinase-9 and some oncogenes, are grouped in cluster 1. These genes appear to be induced in WKY rats after LA but inhibited in SH and SHHF rats. A few cell-cycle control and growth arrest genes are expressed at lower levels at baseline in SH and SHHF rats compared with WKY saline-treated controls (Figure 5, cluster 2). These genes are inhibited in SH and SHHF rats after LA exposure. Finally, tumor suppressor and growth receptors genes are induced only in WKY rats 3 months after LA exposure (Figure 5, cluster 3).

## Discussion

Asbestos-associated Fe has been postulated to play a role in the inflammatory process; however, its influence on chronic pathology in humans is unknown. The accumulation of Fe at tumor sites has been shown to be a consequence of tumor-associated inflammation and oxidative stress (Kukulj et al. 2010). In contrast, other studies suggest that Fe promotes tumor progression through generation of ROS, leading to cancer through oxidation and damage of DNA (Toyokuni 2011). Recently, we have shown that rat models of CVD-associated Fe overload do not show an exacerbation of acute LA-induced inflammation and furthermore that this acute inflammatory response is reduced by Fe despite its ability to generate ROS in synthetic media (Shannahan et al. 2011a, 2011b).

Here we show that pulmonary fibrosis progresses to a similar degree in all three rat strains over a 3-month period following LA exposure. Fe accumulation in fiber-laden macrophages was primarily noted in SHHF rats with Fe overload. Only SHHF rats developed atypical hyperplastic lesions of bronchiolar origin in response to LA. The overall transcriptional activity supports the development of hyperplastic lesions in SHHF rats after LA exposure as well as the baseline chronic inflammation. However, the lack of persistence in LA-induced inflammatory gene expression despite Fe accumulation in SHHF rats is not consistent with its involvement in LA-induced hyperplastic lesions. Thus, CVD patients with Fe overload might not exhibit significant inflammation with asbestos exposure, while they might be susceptible to developing hyperplastic lesions.

Persistent systemic inflammation and Fe overload are hallmark features of chronic diseases in humans and in animals; however, it is not known if they are consequences of the disease or causal factors (Yao and Rahman 2011). We have shown that SH and SHHF rats have increased baseline pulmonary inflammation and Fe overload that relates to the severity of disease (SHHF > SH) (Shannahan et al. 2010). Genes involved in inflammatory processes mediated by NFκB and other signaling pathways were generally up-regulated at baseline in both SH and SHHF rats compared with WKY rats, along with several metabolic and pathological processes. Thus, systemic chronic inflammation and Fe overload coexist in CVD at baseline.

Gene expression analysis revealed that the LA effect on inflammatory genes that regulate innate immunity is still evident at 3 months postexposure in WKY rats but less so in SHHF and SH rats. Although inflammatory genes were up-regulated at baseline in SH and SHHF rats, many of the genes regulating acquired immunity were generally suppressed (Figure 4, cluster 1). This pattern of response suggests that, at 3 months after LA exposure, SH and SHHF rats are unable to sustain increased inflammatory gene expression despite their elevated baseline lung neutrophils. This may be related to their already high baseline expression and an inability to further up-regulate inflammatory genes after LA exposure.

Persistent inflammatory response in the case of asbestos exposure leads to pulmonary fibrosis at the terminal bronchiolar region, coinciding with the site of fiber deposition (Brody et al. 1981). Despite gene expression pattern differences between strains, our study demonstrates similar degrees of fibrotic lesions in the lung. Even though the LA-induced neutrophilic influx had subsided in all three strains at 3 months, the inflammatory gene expression still remained induced in WKY rats compared with SH and SHHF rats, which express high baseline levels of these genes. Increased inflammatory gene expression may be necessary for progression to fibrosis in all strains regardless of whether gene expression is increased at baseline or after LA exposure. LA-induced inflammation may have been dampened because of accumulated Fe within fiber-laden macrophages in Fe-overloaded SHHF rats. Underlying genetic differences may also be involved in differential regulation of inflammation.

Unlike long thin fibers that remain intercalated at the terminal bronchiolar region, shorter fibers are likely to be readily phagocytosed by alveolar macrophages (Bernstein et al. 2010). *In vitro* studies have shown that even epithelial cells can take up relatively short LA fibers (Duncan et al. 2010). In the present study, light microscopy examinations with phase contrast revealed the presence of LA fibers within alveolar macrophages at 3 months, suggesting that these fibers are taken up by macrophages and not cleared from the lung within this time frame. Amosite fibers similar in length to LA have been shown to be engulfed by macrophages *in vivo* (Bernstein et al. 2010). Additionally, the half-life of these amosite fibers has been shown to be > 90 days, suggesting that some of these fibers will remain in the lung for the lifetime of the animal (Bernstein et al. 2010). The ability of alveolar macrophages to engulf but not clear the LA fibers may result in chronic inflammation and disease progression.

The exacerbated progressive accumulation of Fe within fiber-laden alveolar macrophages in the SHHF rats supports the hypothesis that the increased availability of cellular Fe leads to accumulation of Fe at the site of fiber deposition within the lung. Coating of endogenous Fe on the surface of fibers has been observed in mice and in humans after exposure (Ghio et al. 2006, 2009). Furthermore, our previous study with LA (Shannahan et al. 2011a) and another study with quartz (Ghiazza et al. 2011) demonstrated that the bioavailability of catalytically active Fe may reduce the inflammatory response after fiber exposure. Consequently, the Fe overload in SH and SHHF rats might reduce the inflammatory gene induction caused by LA due to accumulated Fe at the site of fiber deposition.

Surprisingly, only the SHHF rats developed atypical hyperplastic lesions at 3 months after exposure to LA. These proliferative lesions likely originated from bronchiolar epithelial cells and did not involve squamation. Unlike typical hyperplastic changes seen in epithelial cells after asbestos-induced lung fibrosis (Robledo et al. 2000), the hyperplastic changes seen in LA-exposed SHHF rats were classified as atypical based on the stacking of cells, but they were not classified as adenomatous. Such atypical lesions have been noted in bronchoalveolar neoplasia and atypical adenomatous hyperplasia of the lung in humans and have been associated with genetic changes in the epithelial growth factor (*EGF*) receptor (McIntire et al. 2010). EGF receptor expression as evidenced from gene array analysis was up-regulated by LA exposure only in SHHF rats. Case studies with asbestos exposure have shown the coexistence of atypical adenomatous hyperplasia, primary lung adenocarcinoma, and pleural mesothelioma (Tsuzuki et al. 2008). Because these lesions seen in SHHF rats at 3 months did not progress over 6 months, ascertaining their significance is difficult. However, chronic exposure to LA may lead to the development of adenoma in SHHF rats. *TP53* (tumor protein 53) is a key tumor suppressor gene that was induced only in WKY rats exposed to LA. The loss of function of TP53 has been implicated in the development of lung cancer and cellular proliferation after asbestos exposure (Morris et al. 2004). *C-Fos* (*FOS*; *FBJ* murine osteoblastoma viral oncogene homolog)–induced growth factor, a member of the *AP-1* oncogene family implicated in cell motility and invasion (Han et al. 2010), and the *FBJ* (murine osteosarcoma viral oncogene homolog) oncogene were up-regulated only in LA-exposed SHHF rats. The expression of *EGF* receptor implicated in hyperplastic cell growth (Adiseshaiah et al. 2008) was also increased in SHHF rats, as discussed previously. A number of cellular processes indicated significant changes in pathways in cancer and basal cell carcinoma development, especially in SHHF rats. This may facilitate the proliferative phenotype seen in the SHHF rats after LA exposure. Baseline high levels of oxidative stress and inflammation in SHHF rats may also increase their sensitivity to develop proliferative lesions. Thus, the SHHF rat may be an appropriate model to study early predisposition to proliferative changes after asbestos exposure in humans.

Certain limitations of the study should be considered in the interpretation of these findings. First, in addition to Fe overload, the baseline CVD of the SH and SHHF rats may play a role in susceptibility to LA. Second, the use of intratracheal instillation, although allowing precise control for pulmonary dose, might produce a one-time bolus response relative to inhalation that may overwhelm normal repair mechanisms. Third, the temporality of gene expression patterns was not considered, and analysis of whole-lung tissue expression may dilute changes seen at the site of lesion. Follow-up studies using the SHHF rat as a model of pulmonary hyperplasia could be done with microdissection of lesions to analyze unique changes in gene expression of these hyperplastic cells. Finally, because LA has a shorter overall fiber length distribution than other well-studied fibers, the kinetics of retention and effects on pulmonary cells may be different. LA was chosen for this study because of its human relevance; however, future studies are needed to assess the effects of longer fibers in the SHHF rat.

## Conclusion

We report that preexisting host Fe overload, as in the case of CVD, results in progressive accumulation of Fe over time within fiber-laden alveolar macrophages in rats exposed to LA. This accumulation of Fe is not associated with increased LA-induced inflammatory gene expression or fibrosis. Moreover, LA-exposure in SHHF rats is associated with the development of atypical hyperplastic lung lesions of bronchiolar origin. The persistence of LA-induced inflammatory gene expression in WKY rats but not in SH or SHHF rats, together with higher baseline levels of inflammation in the CVD models, indicates that fiber-associated Fe does not play a significant role in chronic inflammation. However, changes in transcriptional activity of genes involved in cell-cycle control, growth, and cancer, along with the development of hyperplastic lesions, may suggest early precancerous effects of LA that exist with CVD-associated Fe overload in SHHF rats and humans.

## Supplemental Material

(496 KB) PDF
