# Supplementary material for: Subchronic Pulmonary Pathology, Iron Overload, and Transcriptional Activity after Libby Amphibole Exposure in Rat Models of Cardiovascular Disease
Source: Environ Health Perspect. 2011 Oct 6;120(1):85–91. doi: 10.1289/ehp.1103990 (PMC3261949; doi:10.1289/ehp.1103990)
Supplement: (496 KB) PDF [file ehp.1103990.s001.pdf]

Shannahan JH et al., 2011, Supplemental Material

Subchronic Pulmonary Pathology, Iron-Overload, and Transcriptional Activity after Libby Amphibole Exposure in Rat Models of Cardiovascular Disease

Jonathan H. Shannahan,[1] Abraham Nyska,[2] Mark Cesta,[3] Mette C.J. Schladweiler,[4] Beena D. Vallant,[5] William O. Ward,[6] Andrew J. Ghio,[7] Stephen H. Gavett, [4] Urmila P. Kodavanti[4*]

[1] Curriculum in Toxicology, UNC School of Medicine, Chapel Hill, NC 27599, USA

[2] Consultant in Toxicologic Pathology and Sackler School of Medicine, Tel Aviv University, Israel, Timrat 36576, Israel

[3] Cellular and Molecular Pathology Branch, National Institute of Environmental Health Sciences, National Institute of Health, Research Triangle Park, NC 27711, USA

[4] Cardiopulmonary and Immunotoxicology Branch, Environmental Public Health Division, National Health and Environmental Effects Research Laboratory, Office of Research and Development, U.S. Environmental Protection Agency, Research Triangle Park, NC 27711, USA

[5] Genomics Core, Research Core Units, National Health and Environmental Effects Research Laboratory, Office of Research and Development, U.S. Environmental Protection Agency, Research Triangle Park, NC 27711, USA

[6] Biostatistics Core, Research Core Units, National Health and Environmental Effects Research Laboratory, Office of Research and Development, U.S. Environmental Protection Agency, Research Triangle Park, NC 27711, USA

[7] Clinical Research Branch, Environmental Public Health Division, National Health and Environmental Effects Research Laboratory, Office of Research and Development, U.S. Environmental Protection Agency, Chapel Hill, NC 27599, USA

Corresponding Author:

Urmila Kodavanti, Cardiopulmonary and Immunotoxicology Branch, Environmental Public Health Division, MD B143-01, National Health and Environmental Effects Research Laboratory, US Environmental Protection Agency, Research Triangle Park, NC 27711. Phone: (919) 541-4963. Fax: (919) 541-0026. Email address: Kodavanti.Urmila@epa.gov

Table of Contents

Subchronic Pulmonary Pathology, Iron-Overload, and Transcriptional Activity after Libby Amphibole Exposure in Rat Models of Cardiovascular Disease 1
Methods.. 4
Libby Amphibole 4
Intratracheal Instillation of Libby Amphibole 4
Cell Differential and Bronchoalveolar Lavage Fluid (BALF) Analysis......................... 5
Lung Histopathology 5
Immunohistochemistry 6
Gene Array... 7
Functional Analysis of Gene Array Dataset 7
Results... 9
Libby Amphibole Characterization.. 9
Pulmonary Inflammation, and Injury as Determined using BALF................................. 9
Figures and Figure Legends.. 111
Supplemental Material, Figure 1.. 111
Supplemental Material, Figure 2.. 13
Supplemental Material, Figure 3.. 14
References... 166

Methods

Libby Amphibole

The Libby amphibole (LA) asbestos sample used in this study was collected from the Rainy Creek Complex near Libby, Montana in 2007 by the United States Geological Survey and was processed to produce inhalable material by Meeker et al., as indicated earlier (Meeker *et al.*, 2003). The sample was further size fractionated by water elutriation as described previously (Webber *et al.*, 2008) in order to isolate a rat respirable fraction ($PM_{2.5}$) using a settling velocity of 3.4×10^{-4} cm s^{-1}. LA fiber size distribution and surface chemical properties have been described in recent publications (Shannahan et al. 2011a; 2011b). The mean fiber length of LA was shorter (~5 μm) relative to other asbestos materials used in many studies.

Intratracheal Instillation of Libby Amphibole

The concentrations of LA selected for intratracheal instillation although high were, in general, comparable to instillation studies using other fiber types (Adamson and Bakowska, 2001, Hirano *et al.*, 1988). Doses were chosen to assure a response in the lung upon instillation allowing for a comparative analysis between strains. Theoretically, a rat will deposit 0.07 mg of fibers during 6 hour inhalation at 10 mg/m^3 based on the assumption that minute volume is 200 ml and the deposition fraction to pulmonary region is 0.10. Intratracheal instillation ensured the delivery of exact concentrations of LA into the lung and allowed us to control for likely strain-related deposition differences due to their variation in breathing parameters (Shannahan *et al.*, 2010). In our previous study approximately 30% of WKY presented with non-pathogenic cardiac hypertrophy

(Shannahan *et al.*, 2010). Therefore WKY group size was increased (n=12) to eliminate the data from those with hypertrophic hearts (heart weight > 1.3g; normal heart weight ~1.1g) and still maintain appropriate group sizes for statistical comparisons.

Cell Differential and Bronchoalveolar Lavage Fluid (BALF) Analysis

Aliquots of BALF were taken for total cell counts (Coulter Inc., Miami, FL, USA), cell differentials, and analyses of lung injury markers. Cell differentials were conducted on Cytospin preparations (Shandon, Pittsburgh, PA), and slides were stained with LeukoStat (Fisher Scientific Co., Pittsburgh, PA). Macrophages and neutrophils were counted under light microscopy and quantified based on total cell count. The remaining cell free BALF was evaluated for the following: total protein (Coomassie plus Protein Assay Kit, Pierce, Rockford, IL), albumin (DiaSorin, Stillwater, MN), and the Fe-binding proteins, ferritin (Kamiya Biomedical Company, Seattle, WA) and transferrin (Trf) (DiaSorin, Stillwater, MN). All assays were adapted for BALF analysis and ran using the Konelab Arena 30 clinical analyzer (Thermo Chemical Lab Systems, Espoo Finland). Data were normalized to volume of BALF.

Lung Histopathology

The left lung from each animal was trimmed, embedded in paraffin, sectioned to a thickness of approximately 3 microns (transverse), and stained with hematoxylin and eosin (H&E), Perls' Prussian blue, or Masson's trichrome. The lung lesions were evaluated with particular attention to the lesion location (e.g., bronchi, terminal bronchioles, alveolar duct, alveoli, interstiteum, centriacinar regions, pleura). The morphological evaluation took into consideration the characteristics of each

inflammatory component, e.g., polymorphonuclear cells, macrophages, fibrosis, microgranulomas, as well as changes in the alveolar epithelium. Lesions were also evaluated for Perls' Prussian blue staining for ferric Fe content and localization and Masson's Trichrome for collagen in order to understand modulation of pulmonary Fe content and progression of fibrosis. Histopathological changes were scored using semiquantitative grading at five levels (0 = normal; 1 = minimal; 2 = mild; 3 = moderate; 4 = severe) taking into consideration the degree of severity and the type of lesion (Shackelford *et al.*, 2002, Nyska *et al.*, 2005). In order to verify pathological changes and possible chronic progression of lesion development in SHHF after LA exposure the study was repeated in 36 additional SHHF rats examining lung pathology 3 months and 6 months post-exposure. SHHF rats were instilled with saline containing 0, 0.25, or 1.0 mg of LA and necropsies were performed 3 or 6 months later for detailed pathological evaluation.

Immunohistochemistry

The cellular origin of hyperplasic changes in SHHF rats was examined immunohistochemically. Five-micron thick lung sections were cut from paraffin embedded lung tissue from rats exposed to 0.0, 0.25 and 1.0 mg LA after 3 months and immunohistochemically stained for several epithelial cell markers. The hydrated lung sections were first incubated with 3% hydrogen peroxide to block endogenous peroxidase. Sections were incubated with primary antibodies specific for selected proteins. Biotinylated secondary antibodies were used with peroxidase-conjugated streptavidin to detect cellular localization of proteins of interest. Positive control slides

using known reactive tissues and negative control slides in which the primary antibody was omitted were also prepared. A board certified pathologist then evaluated the slides.

Gene Array

There were 6 samples per group and gene expression in each sample was assayed on separate chips. Biotin-labeled cRNA was produced from 15 μg total RNA using an Affymetrix IVT-express labeling kit (cat# 901228). Total cRNA was then quantified using a Nano-Drop ND-1000 spectrophotometer (NanoDrop Technologies, Wilmington, DE) and evaluated for quality after fragmentation on a 2100 Bioanalyzer. Following overnight hybridization at 45°C to Affymetrix 230 2.0 GeneChips in an Affymetrix Model 640 GeneChip hybridization oven, the arrays were washed and stained using an Affymetrix 450 fluidics station as recommended by the manufacturer and scanned on an Affymetrix Model 3000 (7G) scanner. After scanning, raw data (Affymetrix .cel files) were obtained using Affymetrix Command Console Operating Software (version 3.0). This software also provided summary reports by which array QA metrics were evaluated including average background, average signal, and 3'/5' expression ratios for spike-in controls, β-actin, and GAPDH.

Functional Analysis of Gene Array Dataset

All DEGs (FDR with significance at < 0.05) were filtered to meet the criteria of intensity greater than or equal to 30 and had a fold change greater than or equal to 1.5 or less than or equal to -1.5. Significant KEGG pathway were identified and among those the pathways involved in inflammation, and cell cycle control were analyzed further at gene expression level. Inflammation genes were assembled from KEGG pathways: 4060,

4062, 4070, 4310, 4510, 4514, 4520, 4620, 4621, and 4670. Cell cycle control genes were assembled from KEGG pathways: 4115, and 5200. For each of the functional lists, genes that were on the pathway-specific list and on the master list were submitted to Eisen's Cluster (Eisen *et al.*, 1998) for hierarchical clustering. The genes were median centered with average linkage. The resulting cluster was displayed using Treeview (Eisen *et al.*, 1998). Two other functional lists, growth and fibrosis, were obtained from NetAffx (http://www.affymetrix.com/estore/analysis/index.affx). Genes that were on both the growth and fibrosis lists were clustered with Eisen's Cluster and displayed with Treeview.

Results

Libby Amphibole Characterization

Fiber characterization parameters were recently published (Shannahan et al. 2011b). In brief, fiber dimensions, of the elutriated LA 2007 sample (fibers with aspect ratio $\geq$ 5) were: mean and standard deviation length 4.99 μm ± 4.53 and width 0.28 μm ± 0.19; median length = 3.59 μm, width = 0.23 μm with upper and lower values of length being 0.52 μm – 27.30 μm and width 0.07 μm – 1.15μm as determined using transmission electron microscopy. The estimated fiber count (fibers with aspect ratio $\geq$ 5) for 1 mg LA sample was 218 x 10^6. In comparison to the elutriated LA 2007 sample used in the present study, air samples from Libby Montana have been shown to contain fibers having a mean length of 7.64 μm ± 8.40 and width 0.51 μm ± 0.46, and median length 5.2 μm and width 0.39 μm with upper and lower values of length being 0.5 μm – 195 μm and width 0.01 μm – 10 μm; when applying an aspect ratio of ≥ 5 (U.S. EPA 2010).

Pulmonary Inflammation, and Injury as Determined using BALF

Baseline levels (saline control) of BALF protein and albumin were significantly higher in SH and SHHF when compared to WKY (SHHF>SH>WKY) (Supplemental Material Figure 1), as evident in our previous study (Shannahan *et al.* 2010). Only SHHF exposed to 1 mg LA demonstrated elevated BALF protein at 3 months, however, because of the variable and high levels at baseline, it was difficult to ascertain if this increase was related to LA exposure. The recovery of alveolar macrophages in BALF was unchanged

after LA exposure in all strains despite increased histological cellularity and presence of fiber-laden macrophages. As seen at earlier time points, neutrophils were significantly increased at baseline in SHHF compared to SH and WKY while LA-induced increases in neutrophils noted 1 day through 1 month (Shannahan et al., 2011a) time points, were largely reversed by the 3 months in all strains. Baseline levels (saline control) of BALF ferritin and transferrin were significantly higher in SHHF when compared to WKY (SHHF>SH>WKY) (Supplemental Material Figure 1) suggesting an underlying state of CVD associated Fe-overload. However, at 3 months LA-induced increases noted during earlier time points (Shannahan et al. 2011b) were largely reversed.

Figures and Figure Legends

Supplemental Material, Figure 1

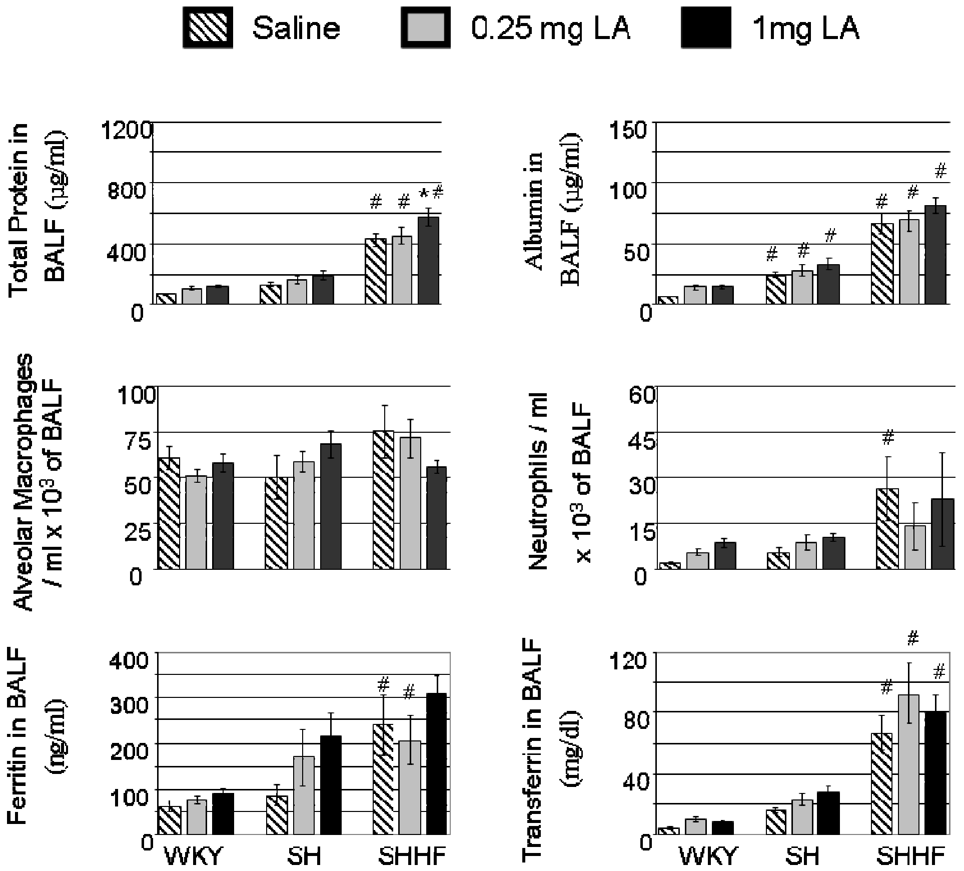

Supplemental Material, Figure 1 Legend: Alterations in BALF protein, albumin, macrophages, neutrophils, ferritin, and transferrin in WKY, SH and SHHF rats at 3-months following intratracheal instillation of saline (control), 0.25 mg LA, or 1 mg LA. Values are mean ± SE (WKY, n =8-12/group; SH and SHHF, n = 6/group). * Indicates

significant difference within strain in respect to saline controls ($p<0.05$). # Indicates significant difference from WKY at the same exposure concentration ($p<0.05$).

Supplemental Material, Figure 2

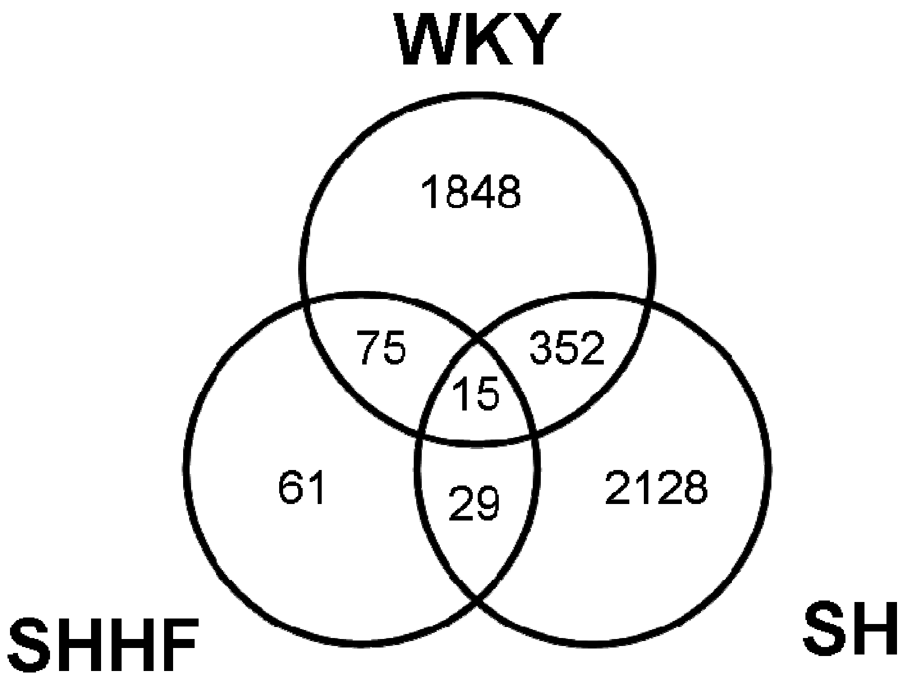

Strain	Total Genes	Up-Regulated	Down-Regulated
WKY	2290	89%	11%
SH	2524	42%	58%
SHHF	180	72%	28%

Supplemental Material, Figure 2 Legend: A Venn diagram of differentially expressed genes (DEGs) comparing WKY, SH, and SHHF rats 3 months after intratracheal instillation exposure to 1.0 mg LA compared to strain matched saline controls. The table shows the direction of changes in DEGs for each strain.

Supplemental Material, Figure 3

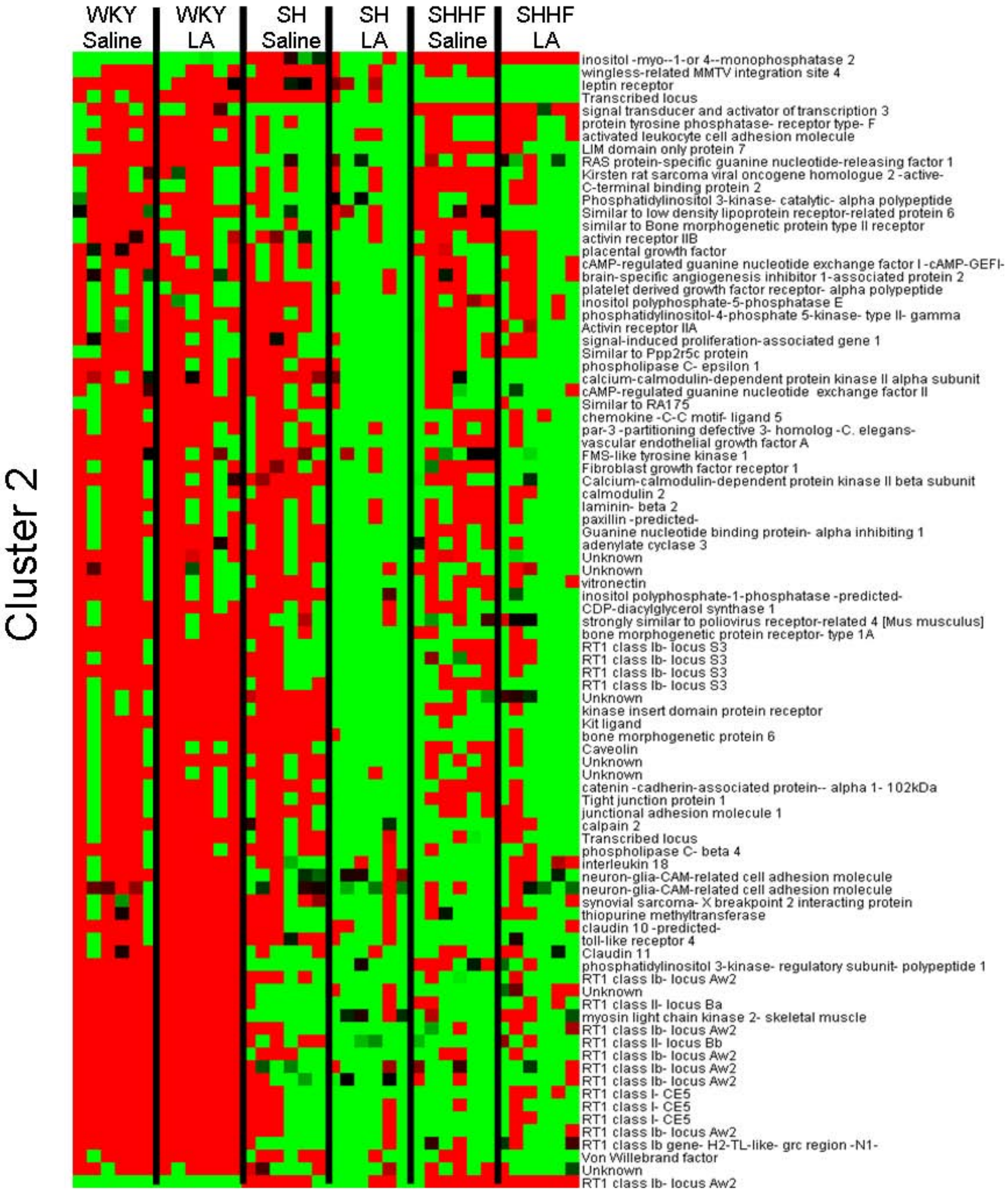

Supplemental Material, Figure 3 Legend: Clustering of inflammation genes comparing WKY, SH and SHHF rats 3-months after intratracheal instillation exposure of

1.0 mg LA. DEGs related to inflammation were assembled from KEGG pathways 4060, 4062, 4070, 4310, 4510, 4514, 4520, 4620, 4621, and 4670. Genes were then hierarchically clustered using Eisen's Cluster Analysis, were median centered with average linkage, and viewed using Treeview. The second cluster (first one is shown in the main manuscript) with genes up-regulated in WKY rats exposed to LA is shown. Red indicates genes that are up-regulated, and green indicates genes that are down-regulated.

References

Adamson, I. Y. and Bakowska, J. (2001). KGF and HGF are growth factors for mesothelial cells in pleural lavage fluid after intratracheal asbestos. *Exp. Lung Res.* **27,** 605-616.

Eisen, M. B., Spellman, P. T., Brown, P. O., and Botstein, D. (1998). Cluster analysis and display of genome-wide expression patterns. *Proc. Natl. Acad. Sci. U. S. A.* **95,** 14863-14868.

Hirano, S., Ono, M., and Aimoto, A. (1988). Functional and biochemical effects on rat lung following instillation of crocidolite and chrysotile asbestos. *J. Toxicol. Environ. Health.* **24,** 27-39.

Meeker, G. P., Bern, A. M., Brownfield, I. K., Lowers, H. A., Sutley, S. J., Hoefen, T. M., and Vance, J. S. (2003). The Composition and Morphology of Amphiboles from the Rainy Creek Complex, Near Libby, Montana. *American Mineralogist.* **88,** 1955-1969.

Nyska, A., Murphy, E., Foley, J. F., Collins, B. J., Petranka, J., Howden, R., Hanlon, P., and Dunnick, J. K. (2005). Acute hemorrhagic myocardial necrosis and sudden death of rats exposed to a combination of ephedrine and caffeine. *Toxicol. Sci.* **83,** 388-396.

Shackelford, C., Long, G., Wolf, J., Okerberg, C., and Herbert, R. (2002). Qualitative and quantitative analysis of nonneoplastic lesions in toxicology studies. *Toxicol. Pathol.* **30,** 93-96.

Shannahan, J. H., Schladweiler, M. C., Richards, J. H., Ledbetter, A. D., Ghio, A. J., and Kodavanti, U. P. (2010). Pulmonary oxidative stress, inflammation, and dysregulated iron homeostasis in rat models of cardiovascular disease. *J. Toxicol. Environ. Health A.* **73,** 641-656.

Shannahan, J., Ghio, A., Schladweiler, M., McGee, J., Richards, J., Gavett, S., Kodavanit, U. (2011a) The role of iron in Libby amphibole-induced acute lung injury and inflammation. *Inhal. Toxicol.* 23, 313-323

Shannahan, J., Schladweiler, M., Padilla-Carlin, D., Nyska, A., Richards, J., Ghio, A., Gavett, S., and Kodavanti, U. (2011b). The role of cardiovascular disease-associated iron overload in Libby amphibole-induced acute pulmonary injury and inflammation. *Inhal. Toxicol.* 23, 129-141

Webber, J. S., Blake, D. J., Ward, T. J., and Pfau, J. C. (2008). Separation and characterization of respirable amphibole fibers from Libby, Montana. *Inhal. Toxicol.* **20,** 733-740.
